# Myosin-binding Protein C Compound Heterozygous Variant Effect on the Phenotypic Expression of Hypertrophic Cardiomyopathy

**DOI:** 10.5935/abc.20170045

**Published:** 2017-04

**Authors:** Julianny Freitas Rafael, Fernando Eugênio dos Santos Cruz Filho, Antônio Carlos Campos de Carvalho, Ilan Gottlieb, José Guilherme Cazelli, Ana Paula Siciliano, Glauber Monteiro Dias

**Affiliations:** 1 Instituto Nacional de Cardiologia; Rio de Janeiro, RJ - Brazil; 2 Casa de Saúde São José, Rio de Janeiro, RJ - Brazil

**Keywords:** Hypertrophic cardiomyopathy, sarcomere genes, compound variant, MYBPC3 gene

## Abstract

Hypertrophic cardiomyopathy (HCM) is an autosomal dominant genetic disease caused by mutations in genes encoding sarcomere proteins. It is the major cause of sudden cardiac death in young high-level athletes. Studies have demonstrated a poorer prognosis when associated with specific mutations. The association between HCM genotype and phenotype has been the subject of several studies since the discovery of the genetic nature of the disease.

This study shows the effect of a *MYBPC3* compound variant on the phenotypic HCM expression.

A family in which a young man had a clinical diagnosis of HCM underwent clinical and genetic investigations. The coding regions of the *MYH7*, *MYBPC3* and *TNNT2* genes were sequenced and analyzed.

The proband present a malignant manifestation of the disease, and is the only one to express HCM in his family. The genetic analysis through direct sequencing of the three main genes related to this disease identified a compound heterozygous variant (p.E542Q and p.D610H) in *MYBPC3*. A family analysis indicated that the p.E542Q and p.D610H alleles have paternal and maternal origin, respectively. No family member carrier of one of the variant alleles manifested clinical signs of HCM.

We suggest that the *MYBPC3-*biallelic heterozygous expression of p.E542Q and p.D610H may cause the severe disease phenotype seen in the proband.

## Introduction

Hypertrophic cardiomyopathy (HCM) is a genetic myocardial disorder characterized by ventricular hypertrophy (VH), which is frequently asymmetrical in the interventricular septum and can lead to a dynamic obstruction of the left ventricle (LV) outflow tract.^1^ It is the main cause of sudden cardiac death (SCD) in young people, with a 2-4% annual mortality rate in adults and 6% in adolescents and children.^2^ A benign outcome of HCM may also occur, such as late onset, mild hypertrophy, and a history of non-malignant events.^3^ Modifier genes, environmental influences, genetic variant diversity and the effect of multiple variants could explain the great clinical heterogeneity between individuals of the same family or from different families.^4^

HCM is a relatively common (0.2%) Mendelian disorder, caused mainly by mutations in sarcomere protein genes, most commonly those encoding β-myosin heavy chain (*MYH7*), myosin-binding protein C (*MYBPC3*) and troponin T (*TNNT2*).^5^ Recent studies suggest that this prevalence is even higher, around 1:200, in the general population,^6^ and around 5% of those who have HCM carry more than one disease-causing gene variant.^7-9^ The hypothesis of gene dosage effects in patients with multiple variants is supported by some authors who have reported a more severe clinical feature, with greater risk of SCD, major LV hypertrophy, and earlier onset of HCM.^7,10^

In this context, we present a case herein in which a compound heterozygous variant led to a HCM manifestation with disease phenotype magnification.

## Methods

### Subjects

The proband with clinical HCM diagnosis was referred to genetic analysis at the National Cardiology Institute (Instituto Nacional de Cardiologia - INC) in Rio de Janeiro. A genealogical tree, including the highest possible number of generations, was built based on his family history. Family members were submitted to clinical assessments and genetic investigations. The local ethics committee approved this study. Written informed consent was obtained for every analyzed family member.

### Clinical assessment

The proband underwent clinical and cardiovascular examination, including a 12-lead electrocardiogram (ECG), transthoracic echocardiography (TTE) and 24-hour Holter monitoring. Diagnosis of HCM was based on TTE: major echo diagnostic criteria were defined by a maximal LV end-diastolic wall thickness ≥ 15 mm. The same clinical examination was performed for the phenotypic analyses of all family members, and cardiac magnetic resonance imaging (CMR) was requested as a complementary exam.

A risk score proposed by the European Cardiac Society (ESC) was used to predict the risk for SCD in five years for patients with HCM.^11^

### Genetic analysis

#### Sanger sequencing

The genetic analysis of the proband was performed through direct sequencing of the three sarcomere genes: *MYH7*, *MYBPC3* and *TNNT2*. Genomic DNA obtained from leukocytes according to Miller et al.^12^ was submitted to a polymerase chain reaction (PCR) of all coding exons, using previously described primers and others designed by us (Tables 1, 2 and 3), and the same amplification program. PCR products were cleaned-up with EXOSAP-IT (Affymetrix, Santa Clara, CA), subjected to the sequencing reaction using the BigDye^®^ Terminator v3.1 reagent (Thermo Fisher Scientific, Waltham, MA) and subsequently analyzed on a ABI 3500xL genetic analyzer (Thermo Fisher Scientific, Waltham, MA). Sequence analyses were performed using the Geneious^®^ v.6.1.6 software package (Biomatters, Auckland, NZ). The family was submitted to a mutation-specific screening according to the HRS/EHRA expert consensus statement.^13^

**Table 1:** Primers for MYH7 sequencing

Exon	Forward Primer 5'-3'	Reverse Primer 5'-3'	Amplicon^†^	A.T.^‡^
3	TCTTGACTCTTGAGCATGGTGCTA	TCTGTCCACCCAGGTGTACAGGTG	381 bp	62ºC
4	AGGAAGGAGGGAAAGCCCAGGCTG	TCTGCATGCACTCAATCTGAGTAA	380 bp	62ºC
5	ATCTTTCTCTAACTCCCAAAATCA	ACTCACGTGATCAGGATGGACTGG	398 bp	60ºC
6	TGTCACCGTCAACCCTTACAAGTG	GAGGCTGAGTCTATGCCTCGGGG	394 bp	62ºC
7	CTTGCTGGTCTCCAGTAGTATTGT	CTGCGGTACAGGACCTTGGAGGGC	198 bp	62ºC
8	GCCCTCCAAGGTCCTGTACCGCAG	GTCCAAGTCCCAAGGCCAAGGTCA	200 bp	62ºC
9	GACAACTCCTCCCGCTTCGTG	AACAGAGGGAGGGAGGGGAGAG	281 bp	62ºC
10	CCTTTTGCTTGCTACATTTATCAT	GCCACAAGCAGAGGGGACCAG	252 bp	60ºC
11	CTGCTTCCTCAGGCCATGTGCTGT	ACCAATGGCCAGAGTCTTAGCTCT	284 bp	62ºC
12	CACAGGGATTAAGGAGACAAGTTT	TTACAGCTGCCCCAAGAATC	273 bp	58ºC
13	AGTCATCTCTTTACCAACTTTGCTA	ATTATCATCTGAAGATGGACCCACC	186 bp	62ºC
14	CAAGTTCACTCTTCCCAACAACCCT	ATGTGGGAGCGAGTGAGTGATTGTT	258 bp	62ºC
15	ACTCACACCCACTTTCTGACTGCTC	GAATTCAGGTGGTAAGGCCAAAGAG	247 bp	62ºC
16	ATAACTGTACTCAGAGCTGAGCCTA	TCCATCCCACTGAGTCTGTAAACCT	578 bp	62ºC
17	GCAAATGCCAGCAAGGATGTAAAG	AGAGAAGGGAGATGGGAAGTAA	359 bp	58ºC
18	CATCTCTGTGACTTCTCGAATTCT	CACTGTGGTGGTAGGTAGGGAGAT	300 bp	60ºC
19	ACAAAGCCAGGATCAGAACCCAGA	GTCCAGAGTCACCCATGCTCTGCA	323 bp	62ºC
20	TGGGTATGAGGGTGCACCAGAGCT	GCATCAGAGGAGTCAATGGAAAAG	330 bp	62ºC
21	TAGGCTGTTACCCTTCCTAAGGTA	GCCTCTGACCCTGTGACTGCAGTG	374 bp	62ºC
22	GGACCTCAGGTAGGAAGGAGGCAG	TGTGCAGGGAGGTGCAGGGTTGTG	390 bp	62ºC
23	TCCTATTTGAGTGATGTGCCTCTC	ATGGTCTGAGAGTCCTGATGAGAC	390 bp	62ºC
24	AGATGGCACCAAGCTGGTGACCTT	TCTGGGCACAGATAGACATGGCAT	290 bp	62ºC
25	GGCAATCTCACAGTCCCCTAATAA	TTTTTGCCAGGGAGGACCATCTAA	508 bp	60ºC
26	ACTCTTTACCTGTATCATTACCAT	GCCTCCATGGACACATAATCAGTT	306 bp	60ºC
27a*	AGCCGAGAGCCTTTTAGAGCCG	GTCCCGCCGCATCTTCTGGA	274 bp	64ºC
27b*	TCCAGAAGATGCGGCGGGAC	AGGGGAGGTGGGAGGAGGAAGT	266 bp	64ºC
28	TCCCACTTCCCTTCCTCTGCCT	CAGCACTCCTCTCTATCCCCACCT	438 bp	56ºC
29	GGTGGGGATAGAGAGGAGTGCTGA	TGTGGCAGGGTTTGGGCTGT	315 bp	64ºC
30	GAGAAGGGCAAGGGTGGGGT	CCTGAGAGGAGAAGGAGGTGGG	422 bp	58ºC
31	TTGTCCCCATCCACACCCTCCA	GCTCCGACTGCGACTCCTCATACT	469 bp	56ºC
32	GCTGAAGAGTGAGCCTTGTCCC	TCCGCTGGAACCCAACTGCT	396 bp	56ºC
33	AGTATGAGGAGTCGCAGTCGGA	GGGGATGAGAACAGGGAGCCAA	500 bp	60ºC
34	CTGCCCTGTGCCCTGACTGT	CCAGCCTCGGTTCCCTTCACT	500 bp	64ºC
35	GTGAAGGGAACCGAGGCTGGC	GTTGGGCAGAGCAGGAAAAGCA	364 bp	62ºC
36	TCCGTGCCAACGACGACCTGAA	GTCCTCACACACTTGCTGCCCA	497 bp	60ºC
37	TGGGCAGCAAGTGTGTGAGGA	GGTTGTCACTGTGGCTATGGTGC	391 bp	62ºC
38 / 39	ACCTTCTATGACTGTGCCATCTTCAC	GTTTGAGGGTGCTCTGTCTGG	464 bp	62ºC
40	ATGCCCTGTCCCTGCCCAATAC	TTTCCACCTCCCCTATGCCAGACC	268 bp	60ºC

(*) Necessary more than one primer pair to cover the exon;

(†) Size of the amplified fragment;

(‡) Annealing temperature

**Table 2:** Primers for MYBPC3 sequencing

Exon	Forward Primer 5'-3'	Reverse Primer 5'-3'	Amplicon*	A.T.^†^
2	GACCTCAGCTCTCTGGAATTCATC	GCTCAGAGGCCACGTCCTCGTCAA	311 bp	62ºC
3	GTGCACGCTCCAACCAG	CAGCAAAGGCAAGAAAGTGTG	429 bp	65ºC
4	CTGGGACGGGGAGGAGAATGTG	GCTTTTGAGACCTGCCCTGGAC	385 bp	62ºC
5	GGGCACCTGCGGTCCCAGCTAACT	ACGCGGGCTGAGAAGGTGATG	378 bp	62ºC
6	CTACCCCTGGAGCCCCCGATGACC	TGCCTCCCAGATTCCCCACACC	449 bp	62ºC
7	CTGGAGCTCCTGGTCTTATGTGAT	GGAGCCGTGACACCAAGATGATAA	528 bp	62ºC
8	GCTTCTCAAACGGCCCCCTCTG	AGCTCCGCCCCGCAAATCATCC	213 bp	62ºC
9	GGGCTGGGGATGATTTG	GGAGGGAGAAAGGGACACTA	226 bp	63ºC
10	AATCTGGCTAGTGTCCCTTTCTCC	AGCCCTTTAACTCCTTCCACACTG	322 bp	62ºC
11	TCGGCCCAACTGACTTA	CCCATGGGCCTTTACTT	389 bp	58ºC
12	CGGCTCCCCACGGACAG	CCCAGGCCAGGCAGGACT	405 bp	67ºC
13	TCCCCAGCCCCTCTTCA	GCCGGACTCCGCTCTTT	515 bp	62ºC
14	GGCGGCACAGAGGGGATTG	ACCGGCAGGAGCAAAAGGATG	402 bp	62ºC
15	ATCCGGCTGACCGTGGAACT	CAGTGCGCCCCGTGATAATC	375 bp	65ºC
16	AACACTTCAACGGCCCCTTCTG	GCCCCCTCCTCCGATACTTCACAC	451 bp	62ºC
17	CGGACGACGCAGCCTACCAGT	GTCAGCTCCACCCCGTCCTTCA	366 bp	62ºC
18	GGAGGAGGGGGCGCAAGTCAAAT	GTCAAAGGCCCAAGGTCACAGAGG	400 bp	62ºC
19	ACAGGCACACGTGTTTTCAC	CAGTCTCCACCTGTCCCATC	345 bp	61ºC
20	AGAATACCAACAAGCCAGGACAAG	GCGGGAAAGTGAGCAGAACC	402 bp	62ºC
21	TGCCTTTGCCCCCGTGCTACTTG	GCCCCAGGACCCCCACTTTTGAT	187 bp	62ºC
22	TCCTCCTGGCTCTCCCGTTTCTCT	GCGCCCCTCTGCTGCTTCTTC	379 bp	62ºC
23	GCTCCTCTGCTCCCTACTTCC	ATGGCCATCAGCACACTTCAC	310 bp	62ºC
24	TCGGTGCCACAGAGATGATTTTGA	GGCTGCCCCTCTGTGTTCTCCA	367 bp	62ºC
25	CCTGTGGCGGTTAGTTGG	CACCGGTAGCTCTTCTTCTTCTTG	350 bp	62ºC
26	CCGAGGGAAGGTGGTGTGG	TCTGTAAAATGCGGCTGAGTATCC	404 bp	62ºC
27	GGAAGTGCCCCCTATGT	TCGCACTGCTCAAAGAAG	457 bp	62ºC
28	TCAGAGGAGTGGGCAGTGGGAGTG	CTGGGGTGTCAATGGCGGGTCTT	292 bp	62ºC
29	GCCTGGAGTTGCTGTGTTAG	GGCTGCCCCTCTTTGGTC	467 bp	62ºC
30	GCGGCCGGCCCTTGGAGT	TGGAAAATGTGAGCTGTGGGTTGG	356 bp	62ºC
31	GCATTCAGGCACTTACCAGGTGACG	CACGGTGAGGACAGTGAAGGGTAGC	527 bp	60ºC
32	GGCCGCAGCTACCCTTCAC	GGCCCCTCTCCCTGTTCC	392 bp	65ºC
33	GGCCTCTCGGTACCAAGTCCTGTC	CAACGTCGGGGCCTGTGAGC	232 bp	65ºC
34	GCAGGGCCATGGTACTCACTCTTG	CCGCCCGCTCTTCCCATCTC	404 bp	62ºC
35	CACAGTGACATGGCCTCCTCTTCT	GCCCCTACAGCCTCCCATTTACT	159 bp	62ºC

(*) Size of the amplified fragment;

(†) Annealing temperature.

**Table 3:** Primers for TNNT2 sequencing

Exon	Forward Primer 5'-3'	Reverse Primer 5'-3'	Amplicon*	A.T.^†^
2	ACAGCTCATGAGGGGTGGAACTA	GTGCTCTGCCTGGGATCTACAACC	376 bp	65ºC
3 / 4	ATGAGAACGGCAGGCCAGGCTAGTG	GTTTGCCTCAAGACCCGAGCAACC	506 bp	65ºC
5	GTGGCGGGAGGTAGCCGACAGT	TGGGCAATCAATGGTTGAATCTTA	403 bp	65º C
6	TTGACCCAGCGCTTCTCTTGTGTC	ACTGGGTGCCACCAATGCAACTTC	449 bp	65º C
7	CCAGTGCCGGGAGGGACTCAC	CAGCCCGTGTCCACTGCACCATAC	262 bp	65º C
8	GGATCAGGGGCCCTGCCTGTCCTGACA	TCCTCCTCCTCTTTCTTCCTGTTCT	538 bp	62º C
9	GCCAGGCCCTGCCAGAGGTCTT	CCCTGGGGGAGGCCTGAAACAG	494 bp	70º C
10	ACGTCCGTGGAGCTGGTTGAAAGT	CCCGGCCAATATTGTCTCTTGACT	373 bp	62º C
11	TGGGAGCTACCCTCTCAGAA	CACAGCAGCTGGGAATCTCT	369 bp	60º C
12	GTAAACCCGGCTGACTACAG	AGCCAGCCCAATCTCTTCAC	258 bp	62º C
13	CAGGGGGTTTGGGGAGGGTTAG	GTGGGGCACCTGCTCAGTTCTCT	402 bp	60º C
14	GGAGGGCCCTTTCTTACTGGAC	CCGGACCCAGTGAACCAGGAGGAG	207 bp	68º C
15	GCCCCTCCTGACCCTTAACTATCC	CGGAGGAGCCAGAGAAGGAAACCT	353 bp	62º C
16	GGGGGTGAAATGTGGGGCGGAGAA	GTGTGGGGGCAGGCAGGAGTGGTG	383 bp	62º C

(*) Size of the amplified fragment;

(†) Annealing temperature.

### Variant pathogenicity prediction

Effects of missense mutations were predicted by using the PolyPhen-2 (http://genetics.bwh.harvard.edu/pph2/), SIFT/PROVEAN (http://SIFT.jcvi.org/) and PredictProtein (http://predictprotein.org/home) tools. A5YM48 and Q14896 were used as *MYBPC3* reference sequences (UniProtKB).

## Results

**Figure 1:**
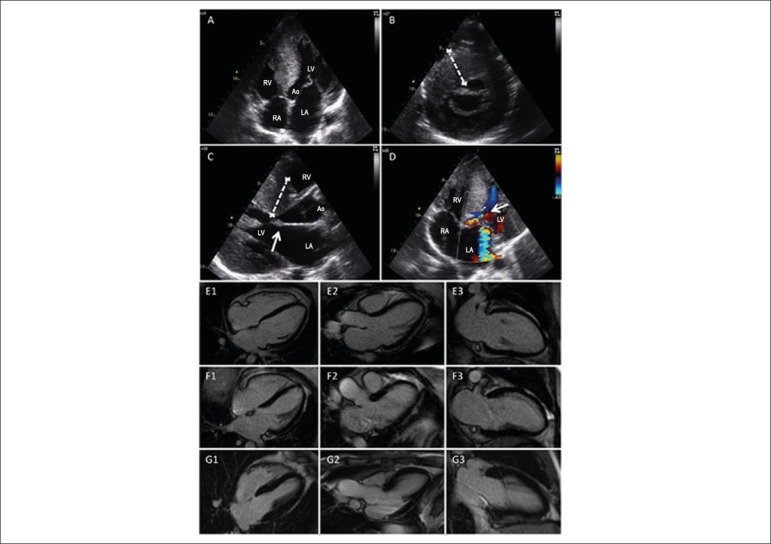
TTE of the proband and CMR of the family. A) TTE image of the four heart chambers and aorta revealing the reverse curve septal hypertrophy. B) Parasternal short-axis view showing the septal hypertrophy. C) Parasternal long-axis view displaying the LV and septal hypertrophy and the enlarged left atrium. The white arrow shows the systolic anterior motion of the mitral valve. D) TTE image showing the obstruction and the turbulence in the outflow tract of the left ventricle (white arrow). Mild mitral regurgitation in the left atrium is visible. CMR of the proband's father (E), aunt (F) and mother (G), showing no hypertrophy or fibrosis signs. CMR in the inversion-recovery sequence (delayed enhancement) in 4CH axes (E1, F1, G1), LVSV (E2, F2, G2) and 2CH (E3, F3, G3). RA: right atrium; RV: right ventricle; LA: left atrium; LV: left ventricle; Ao: aorta.

**Figure 2:**
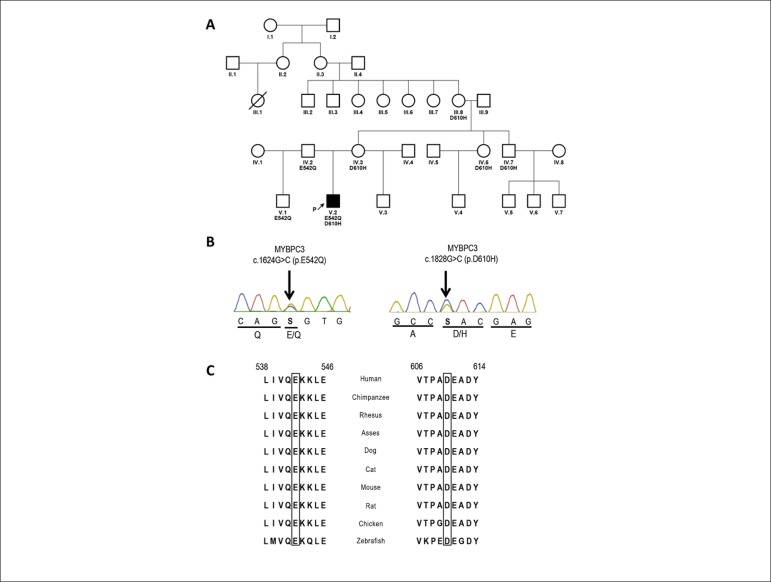
A) Pedigree showing five generations of the maternal family. The proband is the only HCM-affected member. The family variant allele carriers are indicated by E542Q+ and D610H+. B) Electropherograms of the compound missense variant regions of the MYBPC3 gene of the proband. C) Multiple species alignment of the myosin-binding protein C amino acid sequence for residues 538 to 546 and 606 to 614. The conserved residues, glutamic acid and aspartic acid, are indicated by a rectangle.

A seventeen-year-old (y) male proband presenting with a clinical manifestation of HCM and syncope history was submitted to a cardioverter-defibrillator implantation for syncope primary prevention. The diagnosis was based on TTE and showed a reverse curve asymmetric septal hypertrophy, with 39-mm thickness with preserved LV systolic function and normal LV ejection fraction (Figure 1). Additionally, diastolic type II dysfunction, maximum gradient LV/Aorta of 25 mmHg, systolic anterior motion of the mitral valve, obstruction of the LV outflow tract, and enlarged left atrium (46 mm) were also present. The ECG showed LV and LA overload and 24-hour Holter monitoring failed to document the presence of ventricular tachycardia. The risk of SCD was considered high, at 7.69%. The genetic analysis identified a compound heterozygous missense variant, c.1624G>C (p.E542Q) and c.1828G>C (p.D610H) in *MYBPC3* (Figure 2). The variant p.E542Q (rs121909374) has been associated with HCM in ClinVar and in the Human Gene Mutation Database (HGMD). The *in silico* analysis performed by PolyPhen-2 predicts this variant as possibly harmful, while SIFT/PROVEAN and PredictProtein classify this mutation as tolerable. On the other hand, p.D610H (rs371564200) is classified as a variant of uncertain significance (VUS), although pathogenicity prediction tools rank p.D610H as probably deleterious/harmful. Both variants affect conserved residues in the polypeptide chain (Figure 2).

The proband is the only member that manifests the HCM phenotype in his family. His father was adopted, so only maternal ascendants are known. The constructed heredogram revealed 30 relatives, over five generations, in which only one unexplained death of a 30-year-old female with no HCM diagnosis was detected (Figure 2).^14^

Genotyping of maternal family members - grandmother (59y), aunt (29y), uncle (35y) and mother (39y) - detected the p.D610H variant. All family members were asymptomatic, with normal TTE and ECG, with no evidence of VH. On the other hand, the allele p.E542Q was detected in the father (40y) and a paternal sibling (8y), both with normal clinical assessment results (Table 4). CMR was performed in the mother, aunt, and father, and resulted in normal findings, specifically normal LV wall thickness and no signs of fibrosis (Figure 1).

**Table 4:** Clinical assessment data of the individuals

Epidemiology					ECG			TTE								
ID	Age (Y)	Sex	HCM	Variant	LAO	LVO	ABN T wave	LVH +	LVH type	Form	Max LVWT (mm)	LVOG mmHg	LVSD	LVDD	SAM	LA size (mm)
III.8	59	F	No	D610H	No	No	No	No	-	-	10	No	No	No	No	28
IV.2	40	M	No	E542Q	No	No	No	No	-	-	9	No	No	No	No	35
IV.3	39	F	No	D610H	No	No	No	No	-	-	9	No	No	No	No	37
IV.6	29	F	No	D610H	No	No	No	No	-	-	8	No	No	No	No	32
IV.7	35	M	No	D610H	No	No	No	No	-	-	8	No	No	No	No	36
V.1	8	M	No	E542Q	No	No	No	No	-	-	7	No	No	No	No	37
V.2	17	M	Yes	D610H E542Q	Yes	Yes	Yes	Yes	Septal	Reverse Curve	39	25	No	Type I	No	46

The identification numbering (ID) of individuals follows the standard adopted in the pedigree charts (Figure 2); ECG: electrocardiography; TTE: Transthoracic echocardiography; (Y): years; HCM: hypertrophic cardiomyopathy; LAO: left atrial overload; LVO: left ventricular overload; ABN T wave: abnormal T wave; LVH + : left ventricular hypertrophy showed by echo; LVH type: type of the left ventricular hypertrophy; Max LVWT: maximal thickness of the left ventricular wall; LVOG: left ventricular outflow gradient; LVSD: left ventricular systolic dysfunction; LVDD: left ventricular diastolic dysfunction; SAM: systolic anterior motion; LA size: left atrial size.

## Discussion

The present study reports on a young individual with severe HCM who carries a compound *trans*-heterozygous variant in the *MYBPC3* gene, with one allele - p.D610H - inherited from the mother and the other - p.E542Q - inherited from the father. 

Individuals with a single variant did not show any HCM phenotype. The p.E542Q variant, found in the paternal relatives, is associated to HCM, with good prognosis and moderate wall hypertrophy, although only a few studies mentioning this mutation are available^10,15-17^. Pathogenicity prediction of p.E542Q is in agreement with literature data^18-21^.

Moreover, the p.D610H variant, identified in the maternal relatives, also did not manifest any HCM phenotype, even in the oldest investigated familiar member (59y). The association between p.D610H and HCM remains uncertain, despite the fact that pathogenicity predicting tools classified this as probably pathogenic. Only a single study in the literature has identified this mutation, although it did not correlate it with the disease^22^.

In general, a single HCM-heterozygous mutation is sufficient to affect myocardial function and lead to hypertrophy; however, early studies have associated variants in the *MYBPC3* gene with incomplete penetrance, mild VH, low SCD risk and benign clinical evolution^23-25^.

In conclusion, it is suggested that, individually, the p.E542Q and p.D610H variants generate mild changes in protein structure/function, insufficient to cause a strong phenotype. However, the expression of these variants in *trans* may be responsible for early disease onset, a more severe clinical phenotype and increased risk of malignant events in the proband. In other words, double or compound variants by themselves are not decisive for a poorer HCM prognosis, but the allelic composition of these variants may be determinant in this regard.

### Study limitations

The present study investigated the three major HCM-genes that account for approximately 60-70% of HCM cases^5,14^. However, several other genes have already been associated to this disease^5,14^, which are yet to be investigated.
